# Cost-Effectiveness of Anti-Epidermal Growth Factor Receptor Therapy *Versus* Bevacizumab in KRAS Wild-Type (WT), Pan-RAS WT, and Pan-RAS WT Left-Sided Metastatic Colorectal Cancer

**DOI:** 10.3389/fonc.2021.651299

**Published:** 2021-05-03

**Authors:** Shing Fung Lee, Horace C. W. Choi, Sik Kwan Chan, Ka On Lam, Victor H. F. Lee, Irene O. L. Wong, Chi Leung Chiang

**Affiliations:** ^1^ Department of Clinical Oncology, Tuen Mun Hospital, New Territories West Cluster, Hong Kong, Hong Kong; ^2^ Department of Clinical Oncology, University of Hong Kong, Hong Kong, Hong Kong; ^3^ Clinical Oncology Center, The University of Hong Kong-Shenzhen Hospital, Shenzhen, China; ^4^ School of Public Health, University of Hong Kong, Hong Kong, Hong Kong

**Keywords:** colorectal cancer, economic evaluation, decision-making, economic evidence, simulation models

## Abstract

**Objectives:**

We aimed to compare the economic value of chemotherapy plus anti-epidermal growth factor receptor (anti-EGFR) monoclonal antibody (mAb) against chemotherapy with bevacizumab (Bev, an anti-vascular endothelial growth factor mAb) as first-line treatment in KRAS wild-type (WT), pan-RAS WT and pan-RAS WT left-sided metastatic colorectal cancer (mCRC) patients from the Hong Kong societal perspective.

**Materials and Methods:**

We developed Markov models and 10-year horizon to estimate costs, quality-adjusted life years (QALYs), and incremental cost-effectiveness ratio (ICER) of chemotherapy plus anti-EGFR therapy against chemotherapy plus Bev in KRAS WT, pan-RAS WT, and pan-RAS WT left-sided mCRC. We considered two times of the local gross domestic product per capita (GDPpc) as the willingness-to-pay (WTP) threshold (2× GDPpc; US$97,832).

**Results:**

Adding anti-EGFR mAb to chemotherapy provides additional 0.24 (95% confidence interval [CI] 0.19–0.29), 0.32 (95% CI 0.27–0.37), and 0.57 (95% CI 0.49–0.63) QALY compared to adding Bev in KRAS WT, pan-RAS WT, and left-sided pan-RAS WT mCRC populations respectively. The corresponding ICER is US$106,847 (95% CI 87,806–134,523), US$88,565 (95% CI 75,678–105,871), US$76,537 (95% CI 67,794–87,917) per QALY gained, respectively.

**Conclusions:**

Anti-EGFR therapy is more cost-effective than Bev as a first-line targeted therapy in left-sided pan-RAS WT and pan-RAS WT, with ICER <US$100,000/QALY, compared to KRAS WT mCRC population.

## Introduction

Colorectal cancer (CRC) is a significant global health burden. Over the past decades, the introduction of molecular targeted therapy has dramatically improved the prognosis of metastatic colorectal cancer (mCRC) patients, with their median survival doubled from 14–16 months to over 30 months (1–5). Combination chemotherapy plus targeted therapy, either anti-epidermal growth factor receptor (anti-EGFR) monoclonal antibody (mAb) or anti-vascular endothelial growth factor (anti-VEGF) mAb have become the current standard first-line treatment.

Both anti-EGFR mAb and bevacizumab (Bev, an anti-VEGF mAb) have demonstrated their efficacies as first-line therapies in KRAS wild-type (WT) patients. However, three randomized trials of head-to-head comparisons between the two agents showed conflicting results (4–6). The CALGB 80405 trial, which is the largest one, has demonstrated equivalence of anti-EGFR mAb and bevacizumab in terms progression-free survival (PFS) and overall survival (OS) (5). However, both the FIRE3 and the PEAK studies have suggested the superiority of anti-EGFR therapy (4, 6). Definitive evidence in supporting one agent remains lacking; therefore, authorities recommended both agents as the acceptable options (7, 8). However, post-hoc analyses suggested that the benefit of anti-EGFR therapy is more pronounced in pan-RAS WT patients (9, 10). Recently, the primary tumor location (PTL) has been validated as a response predictor of anti-EGFR mAb, whose benefit is mainly seen in patients of left-sided but not right-sided colonic tumors (10–12).

Economic modeling is one of the frameworks to compare the benefit of different therapeutic options. Although previous studies have shown conflicting results on the value of anti-EGFR mAb as treatment of mCRC, with improvement in patient selection based on biomarkers, we hypothesized that anti-EGFR mAb would be a cost-effective treatment in the biomarker-enriched population. As such, we conducted cost-effective analyses to compare anti-EGFR mAb versus bevacizumab in KRAS, pan-RAS WT patients, and the subgroup of left-sided pan-RAS WT tumor.

## Materials and Methods

### Model Overview

**Figure 1:**
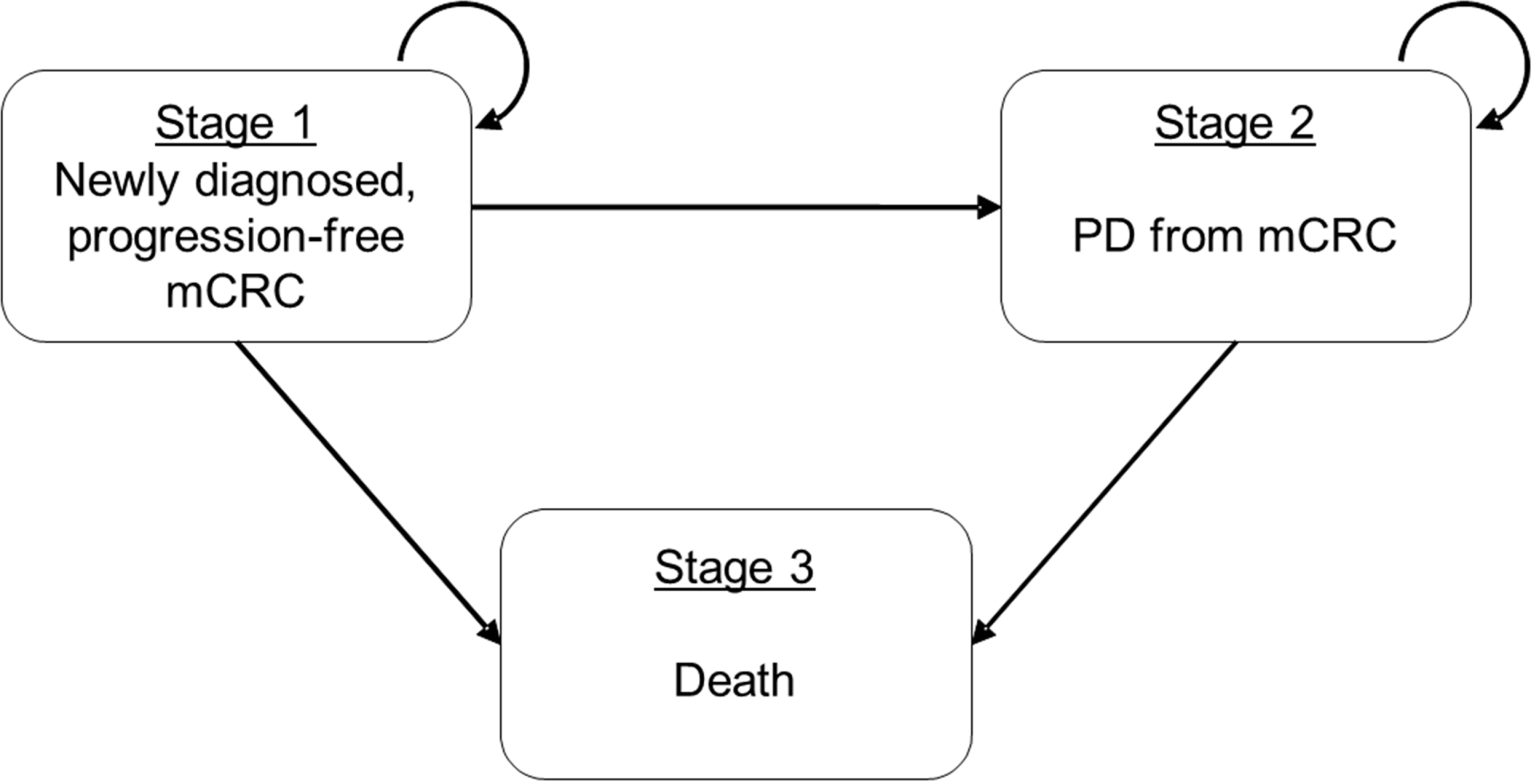
Schematic presentation of three-state Markov model for mCRC. Simplified Schematic the three-state Markov transition model on metastatic colorectal cancer (mCRC). States 1 and 2 (mCRC and PD, respectively) are the recurrent states that patients may stay at the same state in the next time step and State 3 (Death) is the absorption state. mCRC, metastatic colorectal cancer; PD, progressive disease.

We developed a three-state Markov model to analyze the cost-effectiveness of first-line mCRC management from Hong Kong’s societal perspective (**Figure 1**). The entire economic evaluation used data from published studies and was exempted from institutional review board approval.

We reviewed standard literature database (PubMed, Cochrane library, ASCO and ESMO congress database). Phase II or phase III randomized controlled trials (RCTs) comparing chemotherapy and anti-EGFR mAb versus chemotherapy and bevacizumab as first-line treatment in mCRC patients were selected. Included studies must have survival data available for KRAS WT, pan-RAS WT, and pan-RAS WT left-sided tumour populations. pan-RAS genotyping included at least one of the following pan-RAS exons in addition to KRAS exon 2 (codons 12,13): KRAS mutation in exon 3 (codons 59, 61) or 4 (codons 117,146), or NRAS mutations in exon 2, 3 or 4. Based on these criteria, we identified three trials in comparing anti-EGFR mAb versus bevacizumab (FIRE-3, CALGB 80405, and PEAK) (4–6).

We then modeled a hypothetical cohort of patients with KRAS WT mCRC with the same characteristics as those of patients enrolled into the selected RCTs (FIRE-3, CALGB 80405, and PEAK) as the base case (4–6, 10–16). We referred treatment benefits based on survival curves of progression-free survival (PFS) and overall survival (OS) from these trials. The model compared doublet chemotherapy plus anti-EGFR mAb versus doublet chemotherapy plus bevacizumab (Bev). After initial therapy, patients could experience a response and continue therapy, either with or without developing grade 3 or above toxicities, or experience progressive disease (PD) and switch to second-line treatment. Patients on second-line therapy could experience treatment response, or PD, the latter of which would result in termination of active therapy and the commencement of palliative care and death.

### Model Parameterization

In the three-state Markov model (**Figure 1**), namely from progression-free to PD, from progression-free to death, and from PD to death. All transition probabilities for each treatment strategy were estimated based on the PFS and OS curves reported in the RCTs (FIRE-3, CALGB 80405, and PEAK) assessing the respective treatments (4–6, 10–16). The overall estimation process involved two steps: (a) we first estimated the individual patient time-to-event data by reconstructing the reported survival curves of OS and PFS (17); and (b) we then used Markov chain Monte Carlo (MCMC) algorithm to generate the entire parameter sets of transition probabilities. This two-step parameter estimation approach has also been used in our previous cost-effectiveness evaluation of metastatic castration-sensitive prostate cancer (18). We assumed constant transition probabilities between states at each weekly cycle.

### Utilities Estimates

Quality-adjusted life years (QALYs) were calculated by multiplying the time spent in a given state (in life years) by the utility score (a health status value from 0 for death to 1 for perfect health) associated with the corresponding state (19). The utility of all health states and the decrements due to adverse effects (AEs) were derived from the published studies (20–25). We used previously published utilities of 0.72 and 0.63 for patients receiving first-line therapy and second-line therapy respectively (20, 23). We assumed that the utilities for patients who received palliative care would be reduced by half (24, 25). The model considered temporary utility decrements in patients who developed grade 3 or 4 toxicities (22).

### Cost Estimates

Cost parameters included direct and indirect costs. Direct medical costs were drug acquisition, drug administration, and cost for management of AEs. Indirect costs were patients’ time and transportation costs. Costs of chemotherapy, anti-EGFR mAb and bevacizumab were based on the weight or body surface area according to the medication indication (26). Grade 3 or above AEs were included in the model, which composed of acneiform rash, diarrhea, infection, leukopenia, and neutropenia. Management of AEs was based on published guidelines (27). Hospital Authority is the largest healthcare service provider of Hong Kong in taking care of >80% cancer patients in the territory. We referred all medication costs and administration costs to the charges for private service listed in the Government Gazette (28).

### Cost-Effectiveness Analysis

The model outcomes included overall costs (expressed in US Dollar [USD]) and QALYs as the health benefit. We calculated the incremental cost-effectiveness ratio (ICER), which is defined as the incremental difference in costs being divided by the incremental difference in QALYs, to compare cost-effectiveness of treatment strategies. All costs and health outcomes were discounted by 3% annually, with a 10-year time horizon, after which practically all patients have died (29). There is no willingness-to-pay (WTP) threshold suggested by local health authorities in Hong Kong. We considered a more conservative WTP threshold at two times of the local gross domestic product per capita (GDPpc) as the willingness-to-pay (WTP) threshold (2× GDPpc; i.e., US$97,832) (30), which is approximate to the lower limit of the recommendation of US$100,000–150,000 by the Institute for Clinical and Economic Review (31). The model was implemented using the TreeAge Pro 2018 (TreeAge Software, Williamstown, MA) and using R 3.6.1 (R Foundation for Statistical Computing, Vienna, Austria).

### Sensitivity Analysis and Scenario Analysis

We performed a series of sensitivity analyses to explore how results varied across plausible ranges (**Table 1**). The probabilities of developing AEs for each treatment were varied based on beta distribution. Drug costs were varied approximately within 25% of their baseline values. To account for variations in multiple parameters at once, we completed the probabilistic sensitivity analyses; we performed 1,000 Monte Carlo simulations, in which the distributions for all parameters were randomly sampled simultaneously. In one-way sensitivity analyses, we set the value of each parameter at its defined lower and upper extremes and examined the corresponding effect on ICERs. To illustrate the uncertainty, cost-effectiveness acceptability curves were derived and used to project the probability of each treatment strategy to be the most cost-effective under various WTP thresholds. Furthermore, we conducted scenario analyses to evaluate the cost-effectiveness of drugs in pan-RAS WT, and pan-RAS WT left-sided populations.

**Table 1:** Economic and health utility parameters and corresponding distributions for probabilistic sensitivity analysis.

Costs (US$)		One-way sensitivity analysis		Probabilistic sensitivity analysis	
**Systemic Therapy Regime^a^**	**Base value**	**Lower limit**	**Upper limit**	**Distribution^b^**	**Reference**
FOLFOX-4 (every 2 weeks)	219	137	273	*gamma* (100.4, 2.2)	(28)
FOLFIRI (every 2 weeks)	134	87	184	*gamma* (42.5, 3.2)	(28)
mFOLFOX6 (every 2 weeks)	141	93	177	*gamma* (93.4, 1.5)	(28)
XELOX (every 3 weeks)	68	54	83	*gamma* (118.9, 0.6)	(28)
Cetuximab (every 2 weeks)	1577	1183	1972	*gamma* (89.8, 17.6)	(28)
Panitumumab (every 2 weeks)	2318	1738	2897	*gamma* (90.2, 25.7)	(28)
Bevacizumab (every 2 weeks)	1104	828	1380	*gamma* (90.1, 12.3)	(28)
**Treatment of adverse events**	**Base value**	**Lower limit**	**Upper limit**	**Distribution^b^**	**Reference**
Acneiform rash	387	204	569	*gamma* (28.7, 13.5)	(28)
Desquamation	387	204	569	*gamma* (28.7, 13.5)	(28)
Diarrhea	2118	1341	2895	*gamma* (46.0, 46.0)	(28)
Infection	6732	4653	8811	*gamma* (60.4, 111.4)	(28)
Leukopenia	6732	4653	8811	*gamma* (60.4, 111.4)	(28)
Neutropenia	6732	4653	8811	*gamma* (60.4, 111.4)	(28)
**Treatment-related procedures**	**Base value**	**Lower limit**	**Upper limit**	**Distribution^b^**	**Reference**
Laboratory Test	241	181	301	*gamma* (90.8, 2.7)	(28)
Radiographic Test	1494	1256	1731	*gamma* (217.6, 6.9)	(28)
Consultation	192	101	283	*gamma* (28.2, 6.8)	(28)
Hospitalization (per day)	710	568	853	*gamma* (137.1, 5.2)	(28)
Palliative care (per day)	1173	836	1510	*gamma* (69.2, 16.9)	(28)
**Indirect costs**	**Base value**	**Lower limit**	**Upper limit**	**Distribution^b^**	**Reference**
Time cost (per day)	72	51	114	*gamma* (52.5, 1.4)	(21)
Transportation (round trip)	6.4	2.6	10.3	*gamma* (18.4, 0.3)	(21)
**Health utilities**					
**Parameters**	**Base value**	**Lower limit**	**Upper limit**	**Distribution^b^**	**Reference**
Progression-free mCRC	0.72	0.49	0.95	*beta* (17, 6.5)	(20, 23)
Disease progressed mCRC (relative to progress-free mCRC)	0.88	0.75	1	*beta* (42, 5.7)	(20, 23)
Palliative care (relative to progress-free mCRC)	0.50	0.4	0.6	*beta* (67, 66.5)	(24, 25)
Grade 3-4 adverse events (decrement)	0.07	0.0525	0.0875	*beta* (83, 1104.5)	(22)

^a^Calculated based on a weight of 55.6kg and a body surface area (BSA) of 1.6m^2^; costs of chemotherapy preparation and hospitalization stay excluded.

^b^Gamma distribution gamma (shape, scale) assumed for costs and beta distribution beta (α, β) assumed for health utilities.

mCRC, metastatic colorectal cancer.

## Results

### Model Calibration

**Supplementary Figures 1–3** and **Supplementary Table 1** present the comparisons of the model-fitted and the published survival curves for each treatment strategy. The comparisons illustrated a good model fitting for using the estimated parameter sets to reproduce the reported OS and PFS.

### Cost-Effectiveness Analysis

#### Base Case (KRAS WT Population)

Chemotherapy plus anti-EGFR mAb provided an additional 0.24 (95% confidence interval [CI] 0.19 to 0.29) QALY compared with chemotherapy plus Bev (**Table 2**). Their cost incurred were US$128,281 (95% CI 127,397–129,117) and US$153,909 (95% CI 153,011–154,852) respectively. The addition of anti-EGFR mAb to chemotherapy is not cost-effective compared to addition of Bev, with an ICER of US$106,847 (95% CI 87,806–134,523) for each QALY gained under the WTP threshold at US$97,832/QALY (i.e. 2× GDPpc).

**Table 2:** Cost-effectiveness comparison of chemotherapy + anti-EGFR mAb vs. chemotherapy + bevacizumab in (a) KRAS wild-type (b) pan-RAS wild-type and (c) pan-RAS wild-type left-sided colonic tumor.

(a) KRAS wild type	Chemotherapy + bevacizumab^a^	Chemotherapy + anti-EGFR mAb^a,b^
Total discounted cost, US$	128,281 (127,397, 129,117)	153,909 (153,011, 154,852)
Total discounted QALY	1.69 (1.65, 1.72)	1.93 (1.89, 1.96)
		**Chemotherapy + anti-EGFR mAb vs. Chemotherapy + bevacizumab^a,b^**
Incremental discounted cost, US$		25,634 (24,394, 26,870)
Incremental discounted QALY		0.24 (0.19, 0.29)
ICER, US$/QALY		106,847 (87,806, 134,523)
**(b) pan-RAS wild-type**	**Chemotherapy + bevacizumab^a^**	**Chemotherapy + anti-EGFR mAb^a,b^**
Total discounted cost, US$	129,326 (128,473, 130,196)	157,908 (156,818, 158,968)
Total discounted QALY	1.72 (1.68, 1.75)	2.04 (2.00, 2.08)
		**Chemotherapy + anti-EGFR mAb vs. Chemotherapy + bevacizumab^a,b^**
Incremental discounted cost, US$	**-**	28,605 (27,095, 30,053)
Incremental discounted QALY	**-**	0.32 (0.27, 0.37)
ICER, US$/QALY	**-**	88,565 (75,678, 105,871)
**(c) pan-RAS WT left-sided tumor**	**Chemotherapy + bevacizumab^a^**	**Chemotherapy + anti-EGFR mAb^a,b^**
Total discounted cost, US$	138,641 (137,607, 139,716)	181,879 (180,410, 183,369)
Total discounted QALY	1.94 (1.90, 1.98)	2.50 (2.44, 2.56)
		**Chemotherapy + anti-EGFR mAb vs. Chemotherapy + bevacizumab^a,b^**
Incremental discounted cost, US$	–	43,225 (41,421, 45,073)
Incremental discounted QALY	–	0.57 (0.49, 0.63)
ICER, US$/QALY	–	76,537 (67,794, 87,917)

^a^Each cell presents the median and 95% percentile interval among 10,000 probabilistic replications ^b^biweekly 500mg/m^2^ prescription of Cetuximab was assumed following NCCN guideline and local practice in hospitals under the Hospital Authority, Hong Kong.

anti-EGFR mAb, anti-epidermal growth factor receptor monoclonal antibody; QALY, quality-adjusted life year; ICER, incremental cost-effectiveness ratio.

### Scenario Analyses

In the analysis of pan-RAS WT population, chemotherapy plus anti-EGFR mAb provided an additional 0.32 (95% CI 0.27–0.37) QALY compared with chemotherapy plus Bev (**Table 2**). Their cost incurred were US$157,908 (95% CI 156,818–158,968) and US$129,326 (95% CI 128,473–130,196) respectively. Therefore, anti-EGFR is cost-effective compared to Bev, with an ICER of US$88,565 (95% CI 75,678–105,871) for each QALY gained under the WTP at US$97,832 (i.e. 2× GDPpc).

In the analysis of pan-RAS WT left-sided tumors, adding anti-EGFR mAb to chemotherapy provided additional 0.57 (95% CI 0.49–0.63) QALY compared to Bev and led to an ICER of US$76,537 (95% CI 67,794–87,917) per QALY gained (**Table 2**), which was considered to be cost-effective under the WTP threshold at 2× GDPpc. However, in right-sided tumors, using anti-EGFR mAb provided worse QALY of –0.106 (95% CI –0.390 to 0.094) compared to using Bev.

### Sensitivity and Cost-Threshold Analysis

**Figure 2:**
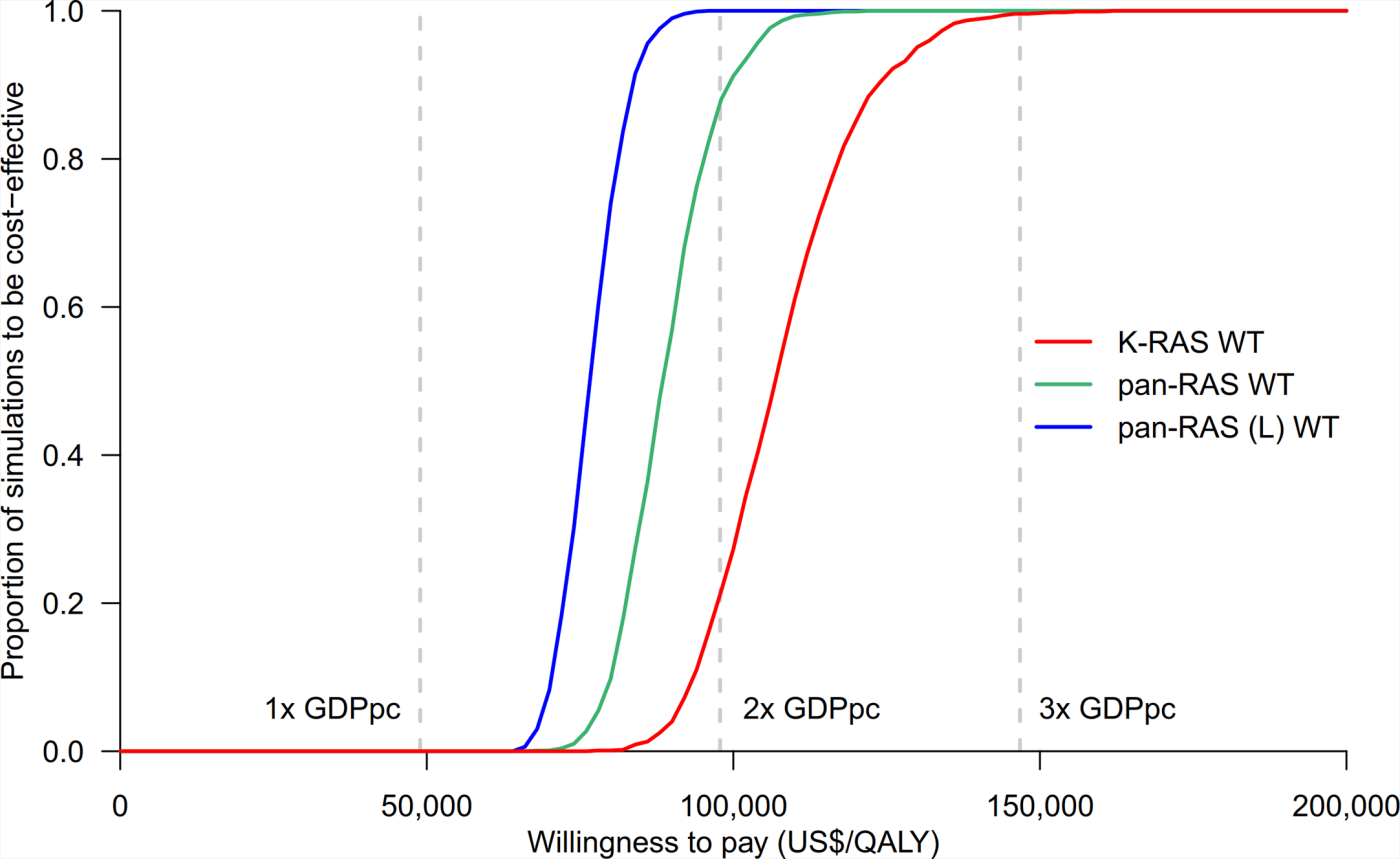
Cost-effectiveness acceptability curves of chemotherapy + anti-EGFR mAb vs. chemotherapy + bevacizumab in KRAS WT, pan-RAS WT, and pan-RAS WT left-sided colonic tumor. anti-EGFR mAb, anti-epidermal growth factor receptor monoclonal antibody; GDPpc, gross domestic product per capita; QALY, quality-adjusted life years; pan-RAS (L) WT, pan-RAS wild-type left-sided colonic tumor; WT, wild-type.

**Figure 3:**
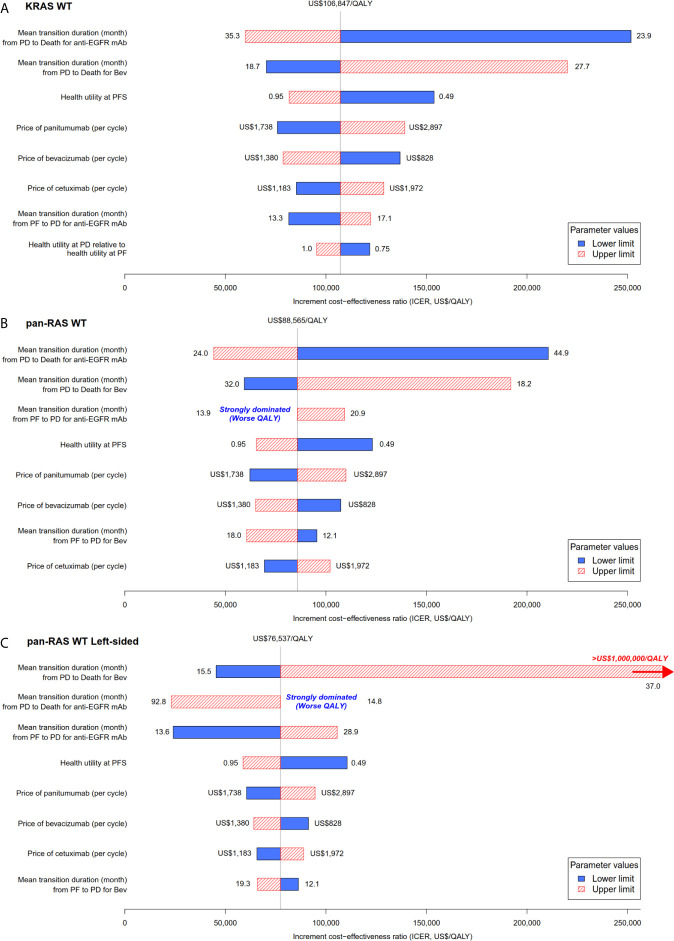
Tornado plot for the one-way univariable sensitivity analyses: chemotherapy + anti-EGFR mAb vs. chemotherapy + bevacizumab in **(A)** KRAS WT, **(B)** pan-RAS WT, and **(C)** pan-RAS WT left-sided colonic tumor. anti-EGFR mAb, anti-epidermal growth factor receptor monoclonal antibody; PD, progressive disease health state; PF, progression-free health state; PFS; progression-free survival; QALY, quality-adjusted life years; WT, wild-type.

**Figure 4:**
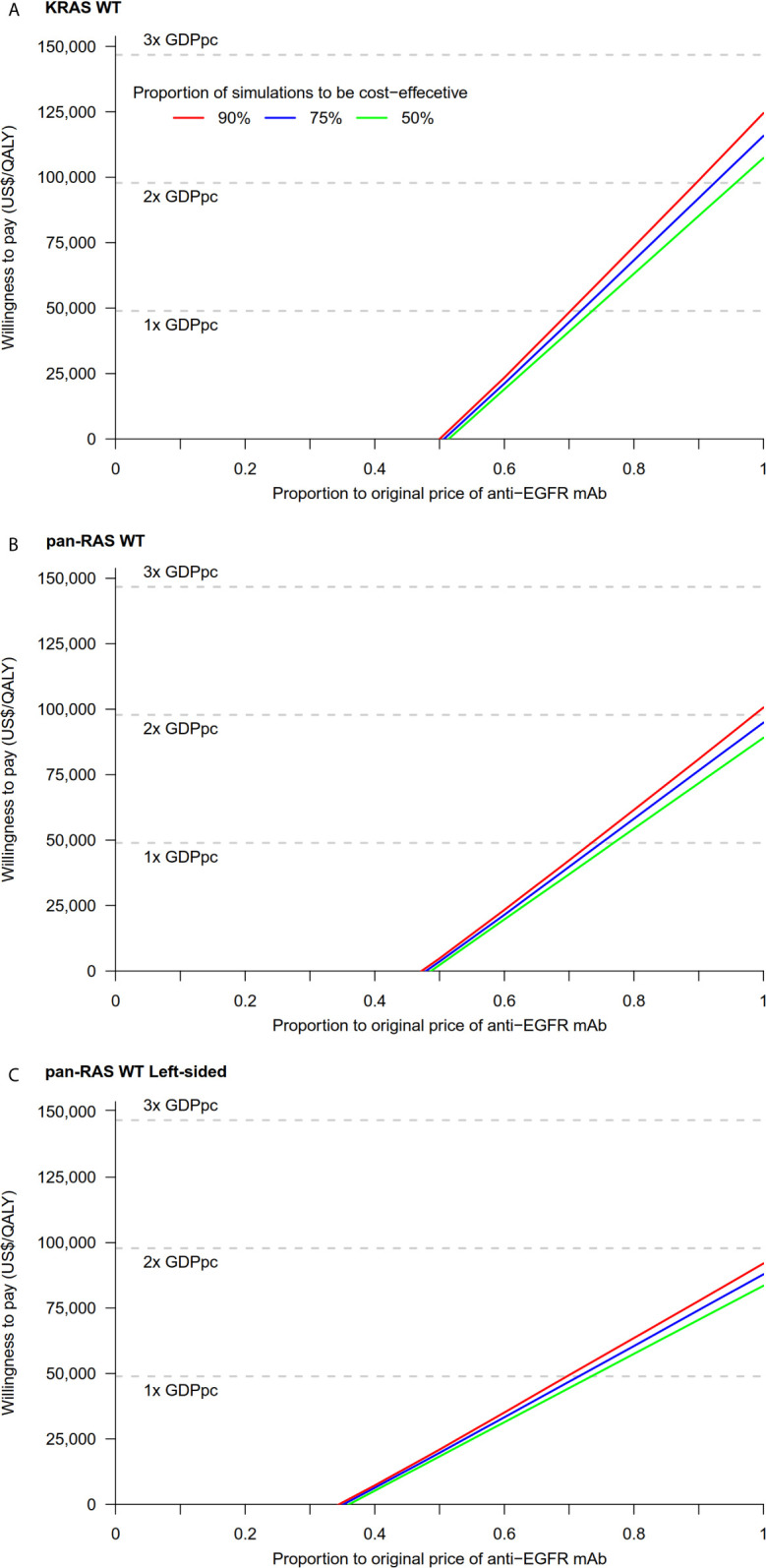
Cost-threshold analyses: chemotherapy + anti-EGFR mAb vs. chemotherapy + bevacizumab in **(A)** KRAS WT, **(B)** pan-RAS WT, and **(C)** pan-RAS WT left-sided colonic tumor. anti-EGFR mAb, anti-epidermal growth factor receptor monoclonal antibody; GDPpc, gross domestic product per capita; QALY, quality-adjusted life year; WT, wild-type.

Probability sensitivity analysis with Monte-Carlo simulation suggested that anti-EGFR mAb was likely to be cost-effective in pan-RAS WT mCRC patients; 88% of simulations were considered to be cost-effective under the WTP threshold of 2× GDPpc (**Figure 2**). Similar findings were observed in pan-RAS WT left-sided patients (100% of simulations). However, only 22% simulations were cost-effective for KRAS WT patients under the WTP threshold of 2× GDPpc. In one-way sensitivity analyses, the parameters with the most influence on ICER were related to natural progression of survival of mCRC, health utilities, and the prices of the either targeted therapy (**Figure 3**) regardless of patients’ pan-RAS status and PTL. The cost-threshold analysis suggested that 10% reduction in the price of anti-EGFR mAb for it to be cost-effective in 90% of the simulations among KRAS WT mCRC patients, compared to only 1.5% in pan-RAS WT patients (**Figure 4**).

## Discussion

To our knowledge, this study provides one of the most comprehensive assessments to date in evaluating the cost-effectiveness of anti-EGFR mAb as first-line therapy in pan-RAS WT mCRC patients. Our model demonstrated that anti-EGFR therapy is cost-effective compared to Bev in pan-RAS WT in particular left-sided tumor, but not KRAS WT population. Our findings implied treatment selection of mCRC patients based on biomarkers and PTL not only benefit individual’s survival, but also the health care system from value perspective.

Controversies remain the role of prognostic and predictive biomarkers available for selecting patients treated with anti-EGFR mAb. The current standard of care biomarkers for mCRC approved by NCCN included extended RAS (KRAS and NRAS) exons 2, 3, and 4 mutations, BRAF V600E mutation, mismatch repair or microsatellite instability, and HER2 amplification (32). Rarer alterations exist for NTRK fusions, PIK3CA, TP53, and PTEN, however, there is still no consensus on their use in routine clinical practice (33–35). These biomarkers might later be shown to be useful in selecting patients who likely will benefit from anti-EGFR treatments.

Our findings were in contrast with those from the previous studies, which found that anti-EGFR mAb were unlikely to be cost-effective in mCRC in first-line or later line settings. Methodologies varied among studies, with different countries of interest, local drug prices, or treatment line settings (first or later). The clinical trials selected for deriving the base case and the subsequent analyses also differ among studies and variably included FIRE-3, CALGB 80405, PEAK, and other trials (23, 36–40). However, a more plausible explanation is that majority of these studies were conducted in KRAS and unselected population (23, 36–40). Recently, Wong et al. showed that EGFR mAb resulted in QALY gained of 0.226 compared to Bev in left-sided pan-RAS WT mCRC patients and concluded that the selective use of biologics based on PTL was more cost-effective than its unselected usage (39). However, unlike in our study, they did not provide the QALY gained and ICER of anti-EGFR mAb compared to Bev in different scenarios of KRAS WT, pan-RAS WT, and pan-RAS left-sided tumor population to justify their conclusion (39). Also, in this Canadian study, the magnitude of clinical benefit provided by anti-EGFR mAb over Bev could not be offset by their price difference. The variations of local drug price and GDPpc will certainly affect the decision of local authorities on whether anti-EGFR mAb is cost-effective in different countries. However, our conclusion that anti-EGFR mAb would offer its best value for reimbursement in left-sided mCRC pan-RAS WT patients (about 70% of mCRC population) compared with Bev unlikely will be changed (41).

Sensitivity analyses suggested that drug cost is one of the most influential factors of our model. Anti-EGFR mAb is more expensive than Bev and resulted in higher lifetime cost, yet the greater mean clinical benefit of adding anti-EGFR mAb offset its additional cost and yielded an ICER of US$88,565/QALY and US$76,537/QALY in pan-RAS WT and pan-RAS left-side mCRC population respectively, which is well below the WTP threshold of 2× GDPpc at US$97,823. Recent meta-analysis of FIRE-3/AIO KRK0306, CALGB/SWOG 80405 and PEAK studies indicated that patients with pan-RAS WT left-sided mCRC had a significantly greater survival benefit from anti-EGFR treatment compared with bevacizumab treatment, when both were respectively added to standard chemotherapy, with a hazard ratio of 0.71; another analysis found that patients with pan-RAS WT disease tend to have better PFS and OS than KRAS WT patients (42, 43). Yet, in right-sided tumor, anti-EGFR therapy arm induced worse QALY than Bev arm. Our findings were consistent with the international clinical recommendations that cetuximab or panitumumab is the preferred biologic in patients with pan-RAS WT left-sided tumor, while its benefit in right-sided tumors is more controversial (32, 44).

### Strength and Weakness

Our model has several strengths. First, this is the first study to compare the cost-effectiveness of treatment selection based on pan-RAS status and PTL. Our conclusion that anti-EGFR mAb achieved its best value in patients with pan-RAS WT left-sided tumor is likely generalizable across different countries. Secondly, we have included all the prospective large-scale phase II/III RCTs in our model, which avoided the selection bias in choosing the trials in favor of a particular strategy. Previous trials demonstrated a wide range of benefits of anti-EGFR therapy in pan-RAS WT population (4–6, 13–16). Majority of the published cost-effectiveness analyses didn’t include the CALGB-80405 study, which is the largest RCT to date that demonstrated no benefit of anti-EGFR therapy over bevacizumab therapy in KRAS WT population, and only modest survival advantage in pan-RAS WT patients (6). The findings are inconsistent with those shown in FIRE 3/PEAK studies. Our results suggested that anti-EGFR therapy is a cost-effective treatment even if we accounted for the results of CALGB study, based on the good fit between estimated transition probabilities and published outcomes in the current model. Thirdly, the preferences of upfront systemic therapy are different across institutions. For instance, Oxaliplatin-based chemotherapy plus bevacizumab is the preferred first-line regime in United States, while Irinotecan-based regime is more commonly used in Europe (7, 8, 45). The choice of upfront therapy has also impacted on the post-progression therapy. Our model has attempted to account most of the possible first-line regimes and their post-progression therapies accordingly to emulate the clinical practice. Sensitivity analyses suggested the chemotherapy backbone unlikely would affect our model findings.

Our study had several limitations. As with many cost-effectiveness studies, our model was based on retrospective data from previously published studies but not the data prospectively collected. Second, the validity was limited by the availability of data, for example post-progression therapies were not shown in CALGB-80405, OPUS, and Crystal studies (5, 46, 47). There were differences in treatment and patient characteristics across clinical trials that have influenced the model parameters. Thirdly, because of lacking corresponding local data, we used the health utilities estimates from overseas studies. In spite of being used in previous published colorectal cancer models, the utility estimates may not accurately reflect the situations of local population in the present analysis. However, we performed a series of sensitivity analyses to minimize the bias. Fourth, we tried to offer a realistic estimate of the use of treatment in routine clinical practice. However, we used efficacy data from RCTs in which people were younger (median ages ranged from 59 to 65 among FIRE-3, CALGB 80405, and PEAK) and fit (Eastern Cooperative Oncology Groups 0–2), while patients are often older and less fit in real world practice (4–6). Finally, although we attempted to account for the most common clinical scenarios, it is unlikely that we can account for all possible situations. For example, in clinical practice, patients may receive either four-month upfront systemic therapy and change to maintenance therapy, instead of continue therapy until disease progression or unacceptable toxicity; patients could receive singlet or triplet chemotherapy backbone depending on disease burden and treatment tolerance. Also, some patients who respond well to systemic therapy could become eligible for resection of primary and metastatic lesion. We could not emulate all possible conditions. However, these scenarios would not be the usual cases in real world practices.

## Conclusion

The anti-EGFR mAb therapy is a more cost-effective choice than Bev as first-line targeted therapy in left-sided pan-RAS WT, pan-RAS WT population compared to KRAS WT population.

## Data Availability Statement

The original contributions presented in the study are included in the article/**Supplementary Material**. Further inquiries can be directed to the corresponding author.

## Author Contributions

Administrative support: HC. Provision of study materials or patients: CC, SC. Collection and assembly of data: CC, SC, and S-FL. Data analysis and interpretation: SC, CC, and HC. Manuscript writing: all authors. Final approval of the manuscript: all authors. Accountable for all aspects of the work: all authors. All authors contributed to the article and approved the submitted version.

## Funding

This work is supported by the Health and Medical Research Fund, FHB, Hong Kong SAR (HMRF/15161781).

## Conflict of Interest

CC has consulting or advisory role for AstraZeneca and Eiasi; and research funding from AstraZeneca and Merck Kgga unrelated to this study.

The remaining authors declare that the research was conducted in the absence of any commercial or financial relationships that could be construed as a potential conflict of interest.

## References

[1] Siegel RL Miller KD Fuchs HE Jemal A. Cancer Statistics, 2021. CA: A Cancer J Clin (2021) 71(1):7–33. 10.3322/caac.21654 33433946

[2] Cremolini C Schirripa M Antoniotti C Moretto R Salvatore L Masi G. First-line chemotherapy for mCRC—a review and evidence-based algorithm. Nat Rev Clin Oncol (2015) 12(10):607–19. 10.1038/nrclinonc.2015.129 26215044

[3] Weinberg BA Marshall JL Hartley M Salem ME. A paradigm shift from one-size-fits-all to tailor-made therapy for metastatic colorectal cancer. Clin Adv Hematol Oncol (2016) 14(2):116–28. 27057810

[4] Heinemann V von Weikersthal LF Decker T Kiani A Vehling-Kaiser U Al-Batran S-E. FOLFIRI plus cetuximab versus FOLFIRI plus bevacizumab as first-line treatment for patients with metastatic colorectal cancer (FIRE-3): a randomised, open-label, phase 3 trial. Lancet Oncol (2014) 15(10):1065–75. 10.1016/s1470-2045(14)70330-4 25088940

[5] Venook AP Niedzwiecki D Lenz H-J Innocenti F Fruth B Meyerhardt JA. Effect of first-line chemotherapy combined with cetuximab or bevacizumab on overall survival in patients with KRAS wild-type advanced or metastatic colorectal cancer. A randomized clinical trial. JAMA (2017) 317(23):2392–401. 10.1001/jama.2017.7105 PMC5545896 28632865

[6] Schwartzberg LS Rivera F Karthaus M Fasola G Canon JL Hecht JR. PEAK: A Randomized, Multicenter Phase II Study of Panitumumab plus modified Fluorouracil, Leucovorin, and Oxaliplatin (mFOLFOX6) or Bevacizumab plus mFOLFOX6 in patients with previously untreated, unresectable, Wild-Type KRAS Exon 2 metastatic colorectal cancer. J Clin Oncol (2014) 32(21):2240–7. 10.1200/jco.2013.53.2473 24687833

[7] National Comprehensive Cancer Network. NCCN Clinical practice guidelines in oncology. (2019). (Accessed 18 December 2019).

[8] Van Cutsem E Cervantes A Nordlinger B Arnold D. Metastatic colorectal cancer: ESMO Clinical Practice Guidelines for diagnosis, treatment and follow-up. Ann Oncol (2014) 25(suppl 3):iii1–9. 10.1093/annonc/mdu260 25190710

[9] Huxley N Crathorne L Varley-Campbell J Tikhonova I Snowsill T Briscoe S. The clinical effectiveness and cost-effectiveness of cetuximab (review of technology appraisal no. 176) and panitumumab (partial review of technology appraisal no. 240) for previously untreated metastatic colorectal cancer: a systematic review and economic evaluation. Health Technol Assess (2017) 21(38):1–294. 10.3310/hta21380 PMC5512008 28682222

[10] Tejpar S Stintzing S Ciardiello F Tabernero J Van Cutsem E Beier F. Prognostic and Predictive Relevance of Primary Tumor Location in Patients With RAS Wild-Type Metastatic Colorectal Cancer: Retrospective Analyses of the CRYSTAL and FIRE-3 Trials. JAMA Oncol (2017) 3(2):194–201. 10.1001/jamaoncol.2016.3797 27722750 PMC7505121

[11] Boeckx N Koukakis R Op de Beeck K Rolfo C Van Camp G Siena S. Primary tumor sidedness has an impact on prognosis and treatment outcome in metastatic colorectal cancer: results from two randomized first-line panitumumab studies. Ann Oncol (2017) 28(8):1862–8. 10.1093/annonc/mdx119 PMC5834073 28449055

[12] Venook AP Ou F-S Lenz H-J Kabbarah O Qu X Niedzwiecki D. Primary (1°) tumor location as an independent prognostic marker from molecular features for overall survival (OS) in patients (pts) with metastatic colorectal cancer (mCRC): Analysis of CALGB / SWOG 80405 (Alliance). J Clin Oncol (2017) 35(15 suppl):3503. 10.1200/JCO.2017.35.15_suppl.3503

[13] Bokemeyer C Köhne CH Ciardiello F Lenz HJ Heinemann V Klinkhardt U. FOLFOX4 plus cetuximab treatment and RAS mutations in colorectal cancer. Eur J Cancer (Oxford Engl 1990) (2015) 51(10):1243–52. 10.1016/j.ejca.2015.04.007 PMC7508202 25937522

[14] Douillard J-Y Oliner KS Siena S Tabernero J Burkes R Barugel M. Panitumumab-FOLFOX4 treatment and RAS mutations in colorectal cancer. N Engl J Med (2013) 369(11):1023–34. 10.1056/nejmoa1305275 24024839

[15] Maughan TS Adams RA Smith CG Meade AM Seymour MT Wilson RH. Addition of cetuximab to oxaliplatin-based first-line combination chemotherapy for treatment of advanced colorectal cancer: results of the randomised phase 3 MRC COIN trial. Lancet (9783) 2011) 377:2103–14. 10.1016/S0084-3873(11)00195-7 PMC3159415 21641636

[16] Van Cutsem E Lenz HJ Kohne CH Heinemann V Tejpar S Melezinek I. Fluorouracil, Leucovorin, and Irinotecan plus Cetuximab treatment and RAS mutations in colorectal cancer. J Clin Oncol (2015) 33(7):692–700. 10.1200/jco.2014.59.4812 25605843

[17] Guyot P Ades AE Ouwens MJNM Welton NJ. Enhanced secondary analysis of survival data: reconstructing the data from published Kaplan-Meier survival curves. BMC Med Res Methodol (2012) 12:9. 10.1186/1471-2288-12-9 22297116 PMC3313891

[18] Chiang CL So TH Lam TC Choi HCW. Cost-effectiveness analysis of Abiraterone Acetate versus Docetaxel in the management of metastatic castration-sensitive prostate cancer: Hong Kong’s perspective. Prostate Cancer Prostatic Dis (2020) 23:108–15. 10.1038/s41391-019-0161-2 31273290

[19] Neumann PJ Ganiats TG Russell LB Sanders GD Siegel JE. Cost-effectiveness in health and medicine. 2 ed. New York, NY: Oxford University Press (2017).

[20] Meads C Round J Tubeuf S Moore D Pennant M Bayliss S. Cetuximab for the first-line treatment of metastatic colorectal cancer. Health Technol Assess (2010) 14(Suppl 1):1–8. 10.3310/hta14suppl1/01 20507797

[21] Census and Statistics Department. Women and Men in Hong Kong Key Statistics, 2019 edition. Census and Statistics Department, Hong Kong Special Administrative Region (2019).

[22] Fryback DG Dasbach EJ Klein R Klein BEK Dorn N Peterson K. The Beaver Dam Health Outcomes study. Med Decision Making (1993) 13(2):89–102. 10.1177/0272989x9301300202 8483408

[23] Mittmann N Au H-J Tu D O’Callaghan CJ Isogai PK Karapetis CS. Prospective cost effectiveness analysis of cetuximab in metastatic colorectal cancer: evaluation of National Cancer Institute of Canada Clinical Trials Group CO.17 trial. J Natl Cancer Inst (2009) 101:1182–92. 10.1093/jnci/djp232 19666851

[24] Ness RM Holmes AM Klein R Dittus R. Utility valuations for outcome states of colorectal cancer. Am J Gastroenterol (1999) 94(6):1650–7. 10.1111/j.1572-0241.1999.01157.x 10364039

[25] Ramsey SD Berry K Moinpour C Giedzinska A Andersen MR. Quality of life in long term survivors of colorectal cancer. Am J Gastroenterol (2002) 97(5):1228–34. 10.1111/j.1572-0241.2002.05694.x 12017152

[26] Yeo W Lam KO Law ALY Lee CCY Chiang CL Au KH. Adjuvant S-1 chemotherapy after curative resection of gastric cancer in Chinese patients: assessment of treatment tolerability and associated risk factors. Hong Kong Med J (2017) 23(1):54–62. 10.12809/hkmj164885 27966431

[27] Hofheinz R-D Segaert S Safont MJ Demonty G Prenen H. Management of adverse events during treatment of gastrointestinal cancers with epidermal growth factor inhibitors. Crit Rev Oncol/Hematol (2017) 114:102–13. 10.1016/j.critrevonc.2017.03.032 28477738

[28] Hospital Authority. List of private charges Hong Kong Special Administrative Region (2019). Available at: https://www.ha.org.hk/visitor/ha_visitor_index.asp?Content_ID=10045&Lang=ENG http://www3.ha.org.hk/fnc/Pathology.aspx?lang=ENG (Accessed 2019 12 March).

[29] Drummond MF Sculpher MJ Claxton K Stoddart GL Torrance GW. Methods for the economic evaluation of health care programmes. New York, NY: Oxford University Press (2005).

[30] Census and Statistics Department. Table 30: Gross domestic product (GDP), implict price deflator of GDP and per capita GDP(2019). Available at: http://www.censtatd.gov.hk/hkstat/sub/sp250.jsp?subjectID=250&tableID=030&ID=0&productType=8 (Accessed 12 March 2019).

[31] Institute for Clinical and Economic Review. Value assessment framework. https://icer.org/our-approach/methods-process/value-assessment-framework/.

[32] National Comprehensive Cancer Network. NCCN Clinical Practice Guidelines in Oncology: Colon Cancer (version 2.2021). Available at: https://www.nccn.org/professionals/physician_gls/pdf/colon.pdf (Accessed March 17 2021).

[33] Russo A Rizzo S Bronte G Silvestris N Colucci G Gebbia N. The Long and Winding Road to Useful Predictive Factors for Anti-EGFR Therapy in Metastatic Colorectal Carcinoma: The KRAS/BRAF Pathway. Oncology (2009) 77(Suppl. 1):57–68. 10.1159/000258497 20130433

[34] Lo Nigro C Ricci V Vivenza D Granetto C Fabozzi T Miraglio E. Prognostic and predictive biomarkers in metastatic colorectal cancer anti-EGFR therapy. World J Gastroenterol (2016) 22(30):6944–54. 10.3748/wjg.v22.i30.6944 PMC4974592 27570430

[35] Lieu CH Corcoran RB Overman MJ. Integrating Biomarkers and Targeted Therapy Into Colorectal Cancer Management. Am Soc Clin Oncol Educ Book (2019) 39):207–15. 10.1200/edbk_240839 31099678

[36] Riesco-Martínez MC Berry SR Ko Y-J Mittmann N Giotis A Lien K. Cost-Effectiveness Analysis of Different Sequences of the Use of Epidermal Growth Factor Receptor Inhibitors for Wild-Type KRAS Unresectable Metastatic Colorectal Cancer. J Oncol Pract (2016) 12(6):e710–e23. 10.1200/jop.2015.008730 27143148

[37] Hoyle M Peters J Crathorne L Jones-Hughes T Cooper C Napier M. Cost-effectiveness of cetuximab, cetuximab plus irinotecan, and panitumumab for third and further lines of treatment for KRAS wild-type patients with metastatic colorectal cancer. Value Health (2013) 16(2):288–96. 10.1016/j.jval.2012.11.001 23538180

[38] Zhou J Zhao R Wen F Zhang P Tang R Chen H. Economic evaluation study (CHEER-compliant): Cost-effectiveness analysis of RAS screening for treatment of metastatic colorectal cancer based on the CALGB 80405 trial. Med (Baltimore) (2016) 95(27):e3762. 10.1097/md.0000000000003762 PMC5058788 27399059

[39] Wong WWL Zargar M Berry SR Ko YJ Riesco-Martínez M Chan KKW. Cost-effectiveness analysis of selective first-line use of biologics for unresectable RAS wild-type left-sided metastatic colorectal cancer. Curr Oncol (2019) 26(5):e597–609. 10.3747/co.26.4843 PMC6821119 31708653

[40] Graham CN Christodoulopoulou A Knox HN Sabatelli L Hechmati G Garawin T. A within-trial cost-effectiveness analysis of panitumumab compared with bevacizumab in the first-line treatment of patients with wild-type RAS metastatic colorectal cancer in the US. J Med Econ (2018) 21(11):1075–83. 10.1080/13696998.2018.1510409 30091652

[41] Bylsma LC Gillezeau C Garawin TA Kelsh MA Fryzek JP Sangaré L. Prevalence of RAS and BRAF mutations in metastatic colorectal cancer patients by tumor sidedness: A systematic review and meta-analysis. Cancer Med (2020) 9(3):1044–57. 10.1002/cam4.2747 PMC6997095 31856410

[42] Holch JW Ricard I Stintzing S Modest DP Heinemann V. The relevance of primary tumour location in patients with metastatic colorectal cancer: A meta-analysis of first-line clinical trials. Eur J Cancer (Oxford Engl 1990) (2017) 70:87–98. 10.1016/j.ejca.2016.10.007 27907852

[43] Sorich MJ Wiese MD Rowland A Kichenadasse G McKinnon RA Karapetis CS. Extended RAS mutations and anti-EGFR monoclonal antibody survival benefit in metastatic colorectal cancer: a meta-analysis of randomized, controlled trials. Ann Oncol (2015) 26(1):13–21. 10.1093/annonc/mdu378 25115304

[44] Yoshino T Arnold D Taniguchi H Pentheroudakis G Yamazaki K Xu RH. Pan-Asian adapted ESMO consensus guidelines for the management of patients with metastatic colorectal cancer: a JSMO-ESMO initiative endorsed by CSCO, KACO, MOS, SSO and TOS. Ann Oncol (2018) 29(1):44–70. 10.1093/annonc/mdx738 29155929

[45] Van Cutsem E Cervantes A Adam R Sobrero A Van Krieken JH Aderka D. ESMO consensus guidelines for the management of patients with metastatic colorectal cancer. Ann Oncol (2016) 27(8):1386–422. 10.1093/annonc/mdw235 27380959

[46] Van Cutsem E Köhne CH Hitre E Zaluski J Chang Chien CR Makhson A. Cetuximab and chemotherapy as initial treatment for metastatic colorectal cancer. N Engl J Med (2009) 360(14):1408–17. 10.1056/NEJMoa0805019 19339720

[47] Bokemeyer C Bondarenko I Makhson A Hartmann JT Aparicio J de Braud F. Fluorouracil, leucovorin, and oxaliplatin with and without cetuximab in the first-line treatment of metastatic colorectal cancer. J Clin Oncol (2009) 27(5):663–71. 10.1200/jco.2008.20.8397 19114683

