# Supplementary material for: Cost-Effectiveness of Anti-Epidermal Growth Factor Receptor Therapy Versus Bevacizumab in KRAS Wild-Type (WT), Pan-RAS WT, and Pan-RAS WT Left-Sided Metastatic Colorectal Cancer
Source: Front Oncol. 2021 May 3;11:651299. doi: 10.3389/fonc.2021.651299 (PMC8127841; doi:10.3389/fonc.2021.651299)
Supplement: Supplementary file 1 [file DataSheet_1.docx]

Supplementary Material

**Cost-effectiveness of anti-epidermal growth factor receptor therapy versus Bevacizumab in KRAS wild-type (WT), pan-RAS WT, and pan-RAS WT left-sided metastatic colorectal cancer**

**Supplementary Figure 1.** Model calibration against published overall survival and progression-free survival for (a) chemotherapy + bevacizumab (Bev) vs (b) chemotherapy + anti-EGFR mAb among KRAS wild-type colorectal cancer patients.


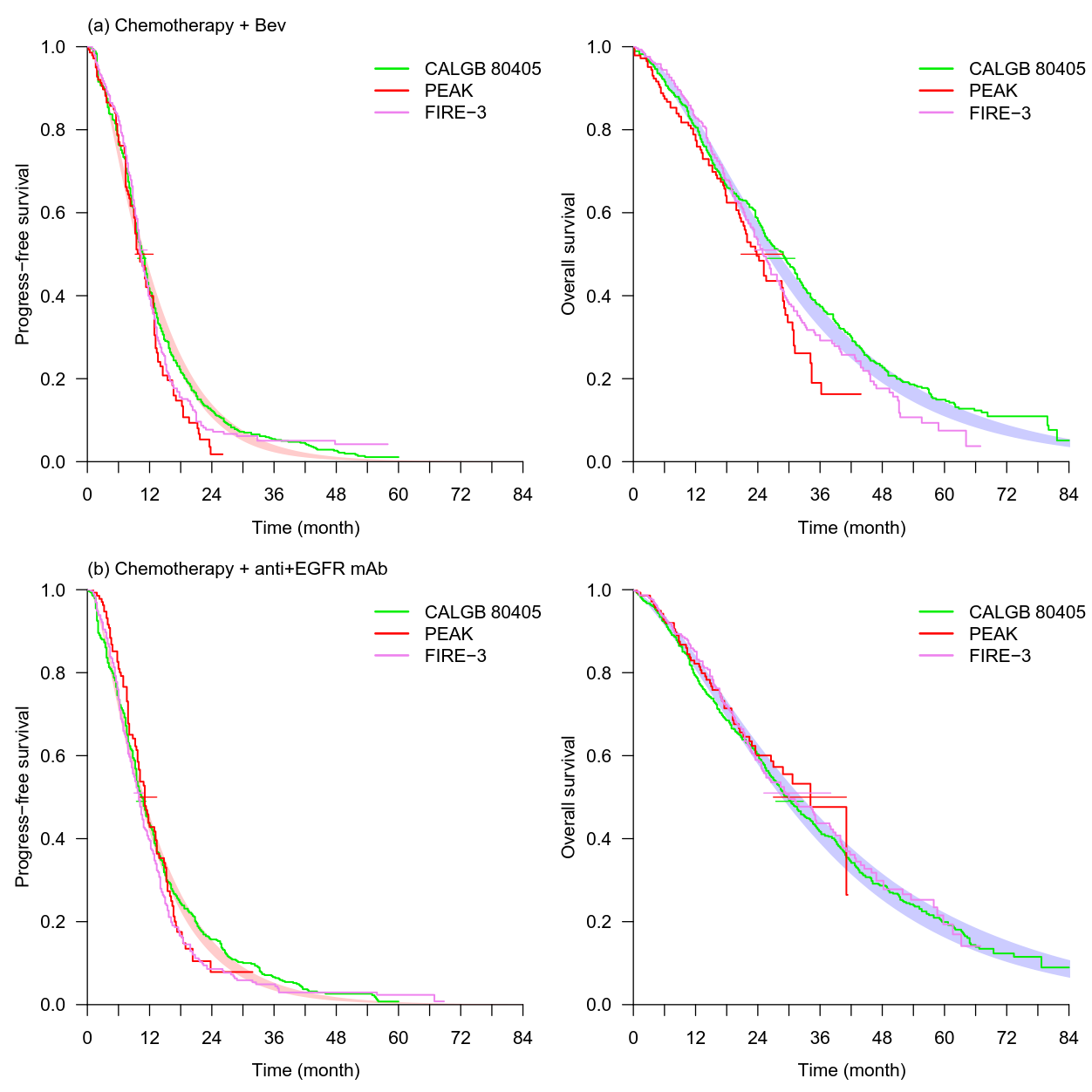


**Supplementary Figure 2.** Model calibration against published overall survival and progression-free survival for (a) chemotherapy + bevacizumab (Bev) vs (b) chemotherapy + anti-EGFR mAb among pan-RAS wild-type colorectal cancer patients.


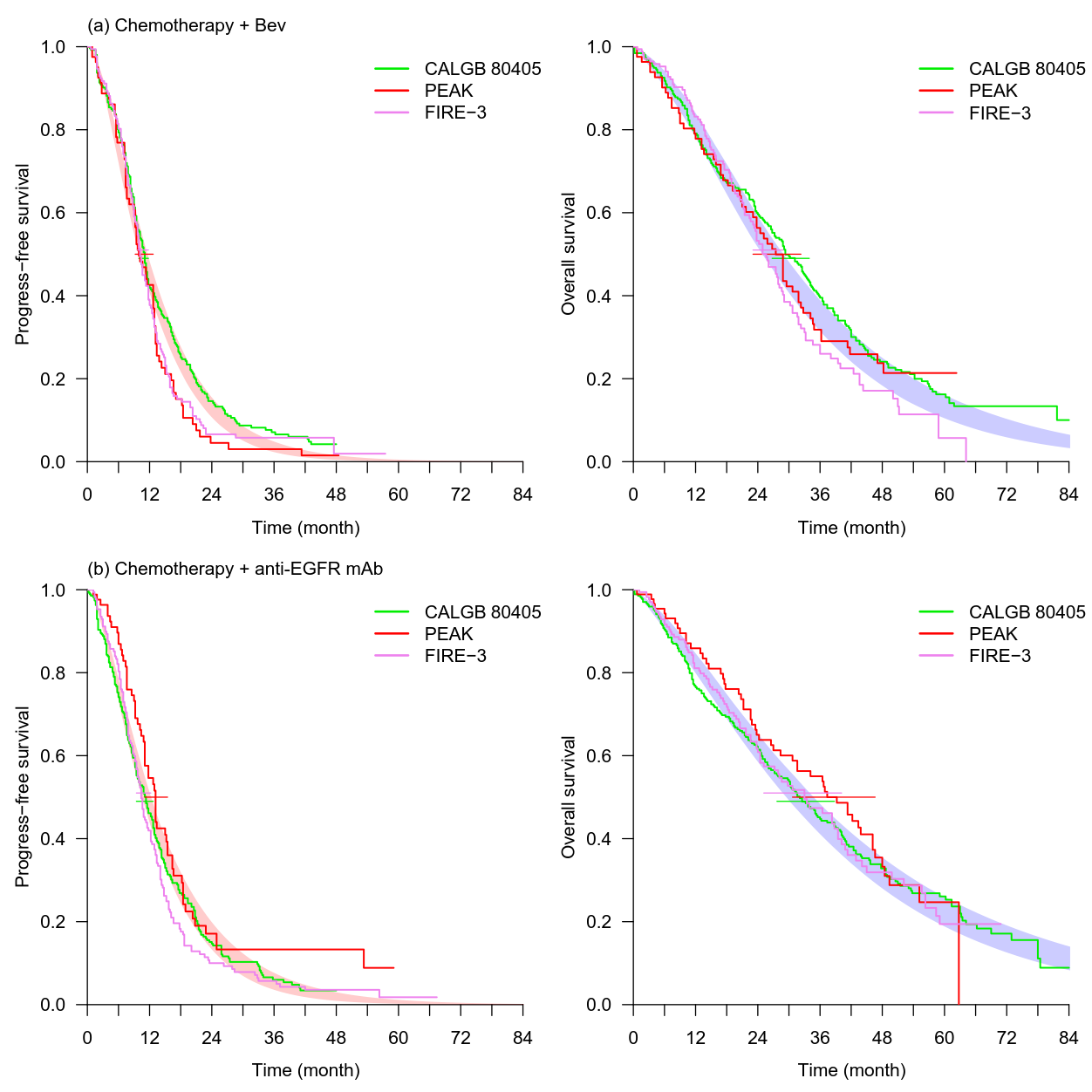


**Supplementary Figure 3.** Model calibration against published overall survival and progression-free survival for (a) chemotherapy + bevacizumab (Bev) vs (b) chemotherapy + anti-EGFR mAb among pan-RAS wild-type left-sided colorectal cancer patients.


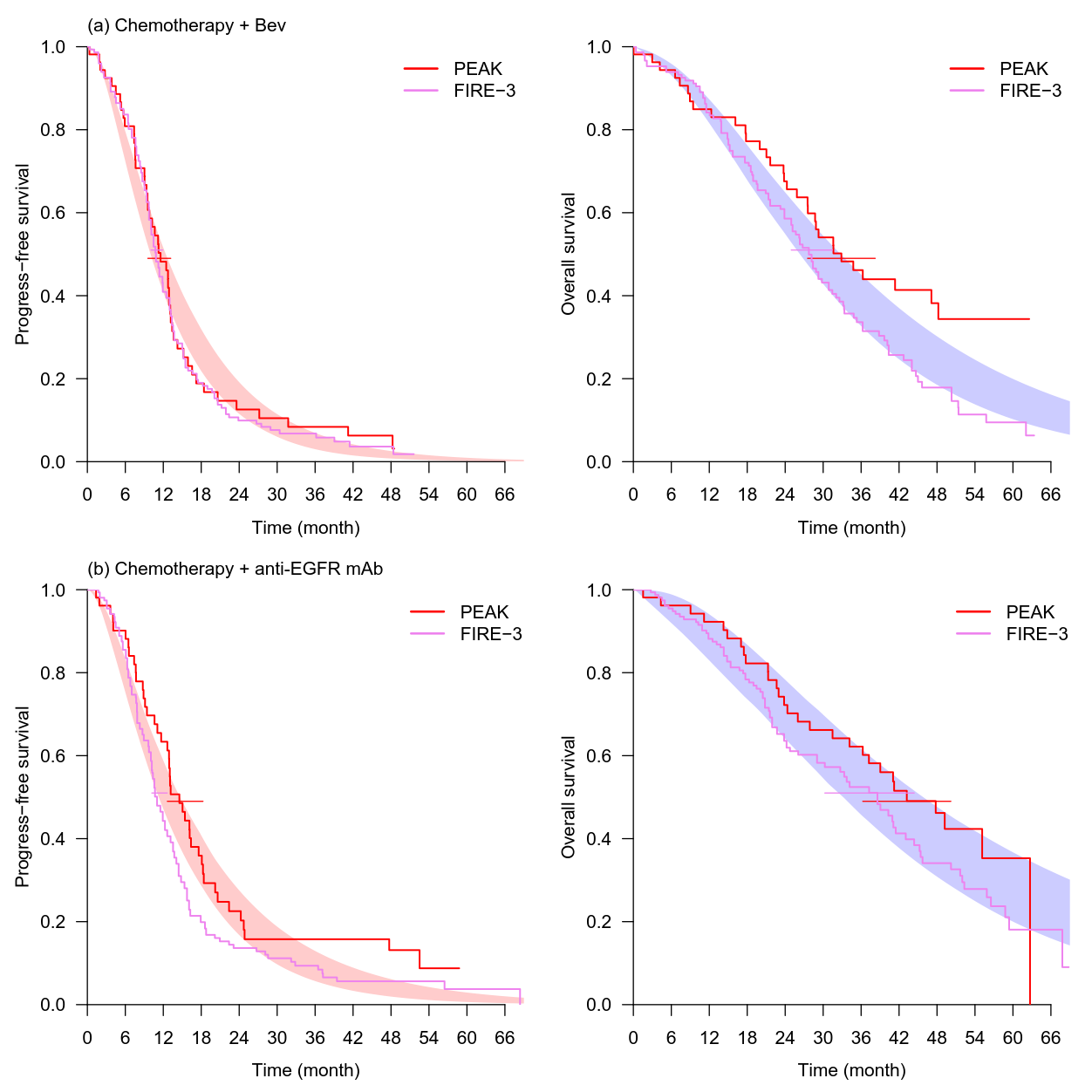


**Supplementary Table 1.** Comparison of median PFS and median OS between model calibration and clinical trials.

|  | Anti-EGFR mAb | | | Bevacizumab | | |
| --- | --- | --- | --- | --- | --- | --- |
| (a) pan-RAS WT* | N | Median PFS (95% CI),^†^month | Median OS (95% CI),^†^ month | N | Median PFS (95% CI),^†^ month | Median OS (95% CI),^†^ month |
| Model calibration | NA | 11.5 (10.6–12.4) | 32.1 (29.6–34.8) | NA | 10.8 (10.2–11.7) | 27.2 (24.9–29.0) |
| PEAK^5^ | 88 | 13.0 (10.9–15.1) | 41.3 (28.8–41.3) | 82 | 9.5 (9.0–12.7) | 28.9 (23.9–31.3) |
| FIRE-3^3^ | 171 | 10.4 (9.5–12.2) | 33.1 (25.4–39.4) | 171 | 10.2 (9.3–11.5) | 25.6 (22.7–28.6) |
| CALGB 80405^4,11^ | 270 | 11.2 (5.9–19.4) | 32.0 (NA) | 256 | 11.0 (7.2–18.2) | 31.2 (NA) |
|  |  |  |  |  |  |  |
| (b) KRAS WT* |  |  |  |  |  |  |
| Model calibration | NA | 10.6 (10.2–11.1) | 30.5 (28.6–32.5) | NA | 10.8 (10.2–11.3) | 26.8 (25.8–28.2) |
| PEAK^5^ | 142 | 10.9 (9.4–13.0) | 34.2 (26.6–NR) | 143 | 10.1 (9.0–12.6) | 24.3 (21.0–29.2) |
| FIRE-3^3^ | 297 | 10.0 (8.8–10.8) | 28.7 (24.0–36.6) | 295 | 10.3 (9.8–11.3) | 25.0 (22.7–27.6) |
| CALGB 80405^4,11^ | 578 | 10.5 (5.8–11.7) | 30.0 (NA) | 559 | 10.6 (6.4–16.6) | 29.0 (NA) |
|  |  |  |  |  |  |  |
| (c) pan-RAS (L) WT |  |  |  |  |  |  |
| Model calibration | NA | 12.9 (11.3–14.8) | 39.5 (33.2–45.9) | NA | 11.3 (10.2–12.5) | 29.8 (26.5–32.9) |
| PEAK^5,10^ | 53 | 14.6 (11.6–18.1) | 43.4 (34.2–63.0) | 54 | 11.5 (9.3–13.0) | 32.0 (26.9–48.5) |
| FIRE-3^3,9^ | 157 | 10.7 (NA) | 38.3 (NA) | 149 | 10.7 (NA) | 28.0 (NA) |
| CALGB 80405^4,11,39^ | 173 | 12.7 (NA) | 39.3 (NA) | 152 | 11.2 (NA) | 32.6 (NA) |

Abbreviations: anti-EGFR mAb, anti-epidermal growth factor receptor monoclonal antibody; CI, confidence interval; NA, not available; NR, not reached; PFS, progression-free survival; OS, overall survival; pan-RAS (L), pan-RAS left-sided colorectal cancer; WT, wild-type.

^*^ Interquartile range (IQR), instead of 95% confidence interval, of PFS were presented for CALGB 80405 trial among pan-RAS WT and KRAS WT populations.

^†^ The cells present the median and corresponding 95% confidence interval (for report of clinical trials) or 95% credible interval (for model calibration) of PFS and OS.
